# A Masterly Analysis

**Published:** 1885-04

**Authors:** 


### A MASTERLY ANALYSIS.

Stapf once consulted Hahnemann about a patient, and mentioned Nux vom., Cham., China, and Puls., as best indicated. Hahnemann analyzed the case in this manner: "Notwithstanding that Nux vom. produced perspiration standing on the forehead, perspiration when moving in general, perspiration during sleep; Chamomilla, perspiration especially about the head during sleep; Pulsatilla, perspiration during sleep, disappearing on awaking; China, perspiration when moving (crying), perspiration on the head especially (but also in the hair);—there is more indication for Pulsatilla by the itching of the eyes, which Puls. has, especially with redness in the *external* corner of the eye after rubbing, and with agglutination of eyelids in morning; if not, Ignatia would be preferable, which also cures itching and redness, but in the *internal* corners, with agglutination in the morning, in case the child's disposition is very changeable—now too lively, next peevishly crying, which Ignatia produces. If there should be, at the same time, a great sensitiveness to the daylight, when opening the eyes in the morning, which is also cured by Ignatia; or, in case of a mild disposition and a weeping mood in the evening, and a general aggravation of symptoms in the evening, Pulsatilla. The frequent awakening during the night indicates Ignatia more than Pulsatilla; the latter has more—a late falling asleep. The itching of the nose has been observed mostly from Nux vomica. Ignatia and Cham. have both, the latter more, pain *during* micturition; Pulsatilla the most pain before urinating. The loud breathing has been observed of China and Nux vom.—from the latter especially during sleep. As these remedies correspond much with each other (China excepted), and one corrects the faults and bad effects of the other (if only Ign. does not follow Nux v., or Nux v. is not given immediately after Ign., as they do not follow one another well on account of their great similarity), you can now judge as to the succession in which you may choose to employ Ign., Puls., Nux v., or Cham.—if the first, or one of the others, should not prove sufficient. To give Cham. there ought to be more thirst at night than at present and more irritability. China has little or nothing for itself, and is therefore not to be chosen."—*Hom. News.*

